# Decline in quality of life among caregivers of patients undergoing chemotherapy for incurable cancer: implications for early social and medical support

**DOI:** 10.1186/s41687-025-00912-2

**Published:** 2025-07-01

**Authors:** Nobumichi Takeuchi, Saiko Kurosawa, Sonomi Yoshida, Kumiko Koike

**Affiliations:** Department of Medical Oncology, Ina Comprehensive Cancer Treatment Centre Ina Central Hospital, 1313 −1 Koshirou-Kubo, Ina, Nagano, 396-8555 Japan

## Abstract

**Background:**

Recent advances in cancer treatment have extended patient survival and improved quality of life (QOL), often enabling home-based chemotherapy. However, this shift places a growing burden on informal caregivers, impacting their own well-being. This study aims to explore changes in caregiver QOL over the course of treatment and identify contributing factors.

**Methods:**

We conducted a single-institution, prospective observational study involving patients receiving chemotherapy for unresectable or recurrent solid tumors and their primary caregivers. QOL was assessed using the EORTC QLQ-C30 before each treatment line. Scores were stratified by treatment duration and line. Paired t-tests and multiple linear regression analyses were performed.

**Results:**

Among 378 patient-caregiver pairs, caregiver emotional and cognitive functioning declined over time, particularly with longer treatment durations and later treatment lines. Fatigue prevalence increased to 100% by the fourth-line treatment. Regression analyses revealed that caregiver QOL was affected by patient symptoms (e.g., insomnia, appetite loss), patient QOL scores, treatment duration, and caregiver age and gender.

**Conclusions:**

Caregivers experienced progressive emotional and cognitive declines paralleling the patient’s clinical trajectory. These findings highlight the necessity of early and comprehensive support systems for caregivers, including psychological and social support, to maintain their QOL throughout cancer treatment.

## Abstract (de)

This study looks at how caring for someone undergoing chemotherapy for advanced cancer affects the caregiver’s well-being. While new treatments help patients live longer and better, caregivers—often spouses or family members—take on more responsibilities at home. These duties can cause stress, fatigue, and social isolation.

We followed over 380 caregiver-patient pairs and found that caregivers’ emotional and cognitive health declined over time, especially as treatment continued or became more complex. Many caregivers reported feeling extremely tired, and some also had trouble sleeping or felt financially strained.

We also found that caregivers’ well-being was influenced by the patient’s symptoms and their own age and gender, with older and female caregivers facing greater challenges.

Our results suggest that caregivers need support from the beginning of cancer treatment, not just near the end. Emotional counseling, social services, and clear information should be offered early to help caregivers stay healthy and able to care for their loved ones.

**Graphical Abstract:**

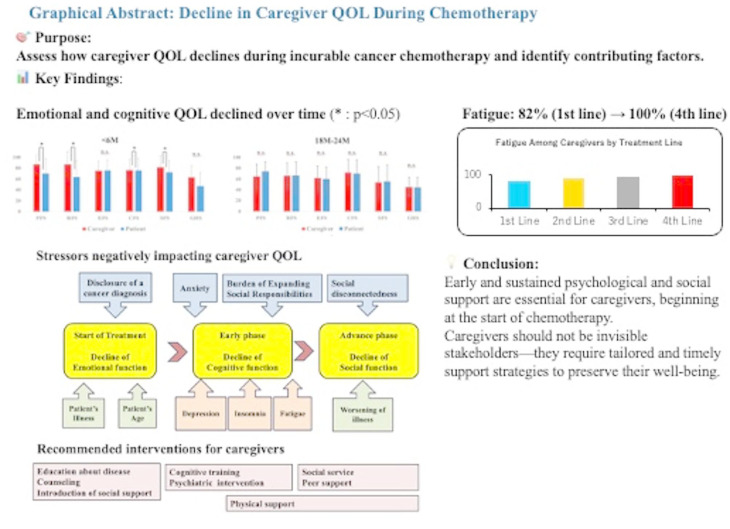

## Introduction

Recent statistics show that over 1 million individuals undergo cancer drug therapy annually in Japan [1], with substantial disparities in treatment access among elderly, rural, and socioeconomically disadvantaged populations [2]. Social determinants of health—including digital literacy, transportation availability, and family structure—play a key role in timely access to care and supportive services for caregivers [3].

This study investigates the decline in caregiver QOL during cancer treatment, hypothesizing that QOL worsens with prolonged treatment and is influenced by patient symptoms and caregiver demographics.

Recent advances in cancer pharmacotherapy have prolonged survival and improved quality of life (QOL) for patients. The use of patient-reported outcomes has enabled clinicians to assess patient’s physical and mental condition more accurately, and maintaining QOL is one of the primary goals of cancer treatment[4–7].

As a result, many chemotherapy regimens are now administered in outpatient clinics, enabling patients to remain at home more often. However, this shift places greater responsibility on caregivers, who must assist with daily tasks and take on medical duties, such as accompanying patients to appointments, managing side effects, and offering emotional support. These responsibilities have raised concerns about the physical, emotional, and social stress placed on caregivers [8, 9]. In addition, caregiving can disrupt work and social activities and contribute to financial hardship due to medical expenses and reduced income[10–12].

Despite ongoing efforts to assist caregivers, evidence on their QOL and firsthand experiences remains limited. Consequently, comprehensive, timely support systems have not been fully developed [10, 13].

In earlier research, we conducted ongoing evaluations of QOL in patients undergoing palliative chemotherapy and their caregivers. The results showed a consistent decline in caregivers’ emotional and cognitive well-being, regardless of treatment duration or efficacy. This highlights the importance of implementing supportive measures early in the treatment process[14]. The aim of this study was to investigate how the quality of life (QOL) of informal caregivers changes during the course of chemotherapy for patients with incurable cancer. We hypothesized that caregiver QOL would progressively decline with prolonged treatment and that this decline would be influenced by patient symptoms, treatment duration, and caregiver demographics such as age and gender. These insights are expected to be useful in determining the optimal timing for caregiver interventions in the future.

## Methods

### Study design

This was a single-institution, prospective observational study.

### Participants

Eligible participants were patients undergoing chemotherapy for unresectable or recurrent solid tumors at our hospital, along with their primary informal caregivers. Inclusion required the patient and caregiver to complete QOL assessments at specific intervals before each line of treatment (from first-line through fourth-line therapy).

Inclusion criteria for patients and caregivers included age ≥ 20 years, Japanese language proficiency, and regular involvement in patient care. Caregivers with cognitive impairment or non-consenting status were excluded.

### Data collection

Data were prospectively collected between August 2016 and March 2024. Before each treatment line, patients and caregivers were informed about the disease and treatment plan, and informed consent was obtained. Self-administered questionnaires were provided to be completed at home or in non-clinical settings without medical personnel present. Questionnaires were collected at the beginning of each treatment cycle.

**Fig. 1:**
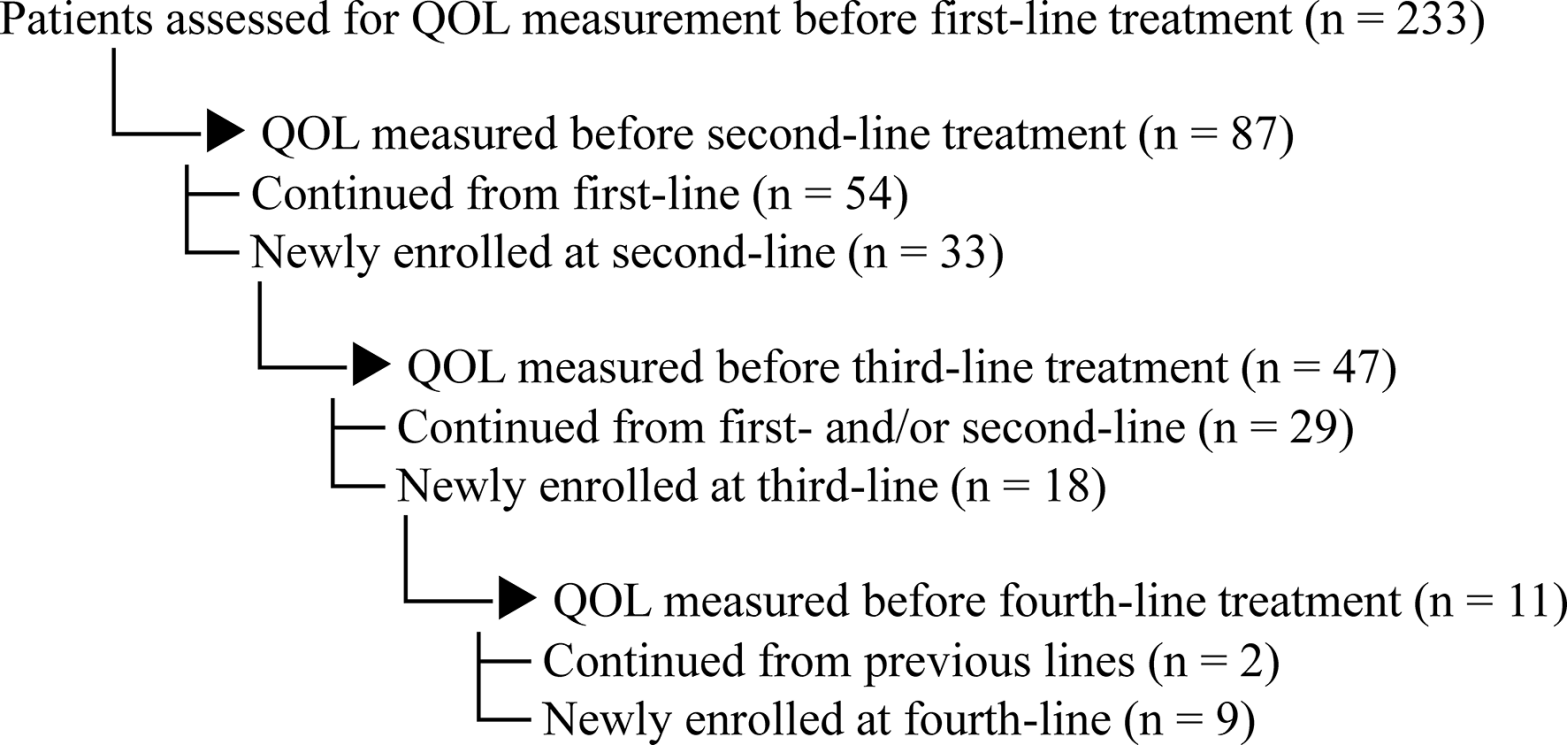
Comparison of QOL Scores Between Patients and Caregivers by Treatment Line. EORTC QLQ-C30 scores for each QOL scale are shown for both patients and caregivers prior to each treatment line (1st to 4th). Error bars represent standard deviation. Note: *p < 0.05, n.s. = not significant

A total of 233 patients were assessed for QOL before first-line treatment. Of these, 54 patients continued to second-line QOL assessment, and 33 additional patients were newly enrolled at the second-line phase. Similarly, 29 patients continued from previous lines to third-line assessment, and 18 new patients were added. In fourth-line treatment, 2 patients continued from prior phases, and 9 additional patients were newly enrolled (Fig. 1).

### Measurement tools

The Japanese version of the EORTC QLQ-C30 used in this study was obtained from the official EORTC Quality of Life Group and has been validated for linguistic and cultural appropriateness in Japanese populations [15].

We used the Japanese version of the EORTC QLQ-C30 (version 3), following the Scoring Manual. The evaluation focused on the following scales: Physical Functioning Scale (PFS), Role Functioning Scale (RFS), Emotional Functioning Scale (EFS), Cognitive Functioning Scale (CFS), Social Functioning Scale (SFS), and Global Health Status (GHS) [16].

### Symptom score definition

Symptom scores from the EORTC QLQ-C30 were calculated by summing individual responses (each rated 1 to 4). Predefined cutoff values were used to determine the presence of specific symptoms. For instance, a total score of 4 or higher indicated fatigue; 3 or higher signified nausea/vomiting and pain; and 2 or higher was used to identify other symptoms.

### QOL stratification

Treatment duration was divided into 6-month intervals, and the average scale scores were compared between patients and caregivers. When multiple assessments fell within the same interval, only the earliest one was included. Similarly, analyses were stratified by treatment line using the same manner.

### Statistical analysis

Paired t-tests were conducted to compare the average scores between patients and caregivers, with a p-value of < 0.05 indicating statistical significance (software: Microsoft Excel for Mac® v16.89.1). To identify factors influencing caregiver QOL, multiple linear regression analysis was performed using (software: SPSS® v30). Independent variables included demographic characteristics of both patients and caregivers such as age, sex, as well as patients’ QOL scores.

### Ethical considerations

The study protocol was approved by the Institutional Review Board of Ina Central Hospital on July 20, 2016 (Approval No. 2016–03). No financial incentives were offered to participants. Informed consent was obtained both in writing and verbally after the study procedures and data collection methods were explained. All data were anonymized.

### Treatment information

Chemotherapy was given either at an outpatient infusion center or in an inpatient ward. In accordance with imaging findings evaluated by RECIST criteria and consideration of treatment-related adverse events, the chemotherapy regimen was modified following a comprehensive discussion with the patient and caregiver [17].

### Additional information

The organization, literature review, and language refinement of this manuscript were partly assisted by ChatGPT-4.0®.

## Results

### Participant characteristics

Table 1 displays the primary cancer site for patients at each treatment line. The demographic characteristics of both patients and caregivers are shown in Table 2. In more than 87% of cases, the caregiver was the patient’s legal spouse (Table 3). The average duration of treatment was 196 days (±137 days), ranging from 45 to 594 days.

**Table 1:**
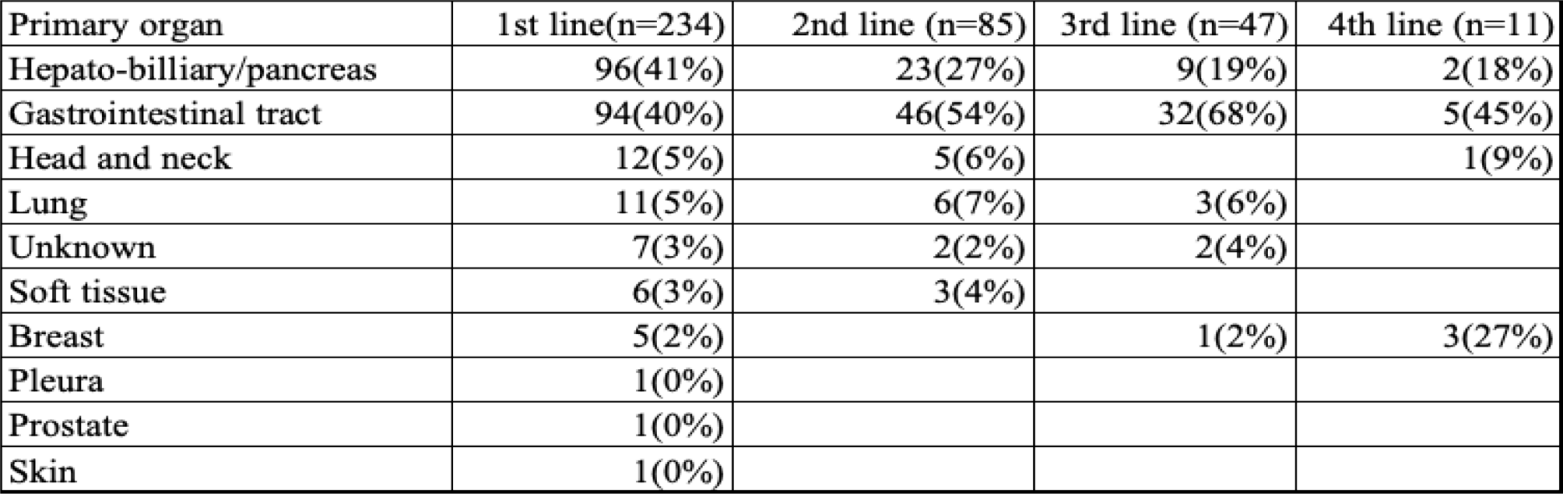
Primary organ of the disease

Legend: This table summarizes the distribution of primary tumor sites among patients receiving chemotherapy at each treatment line

**Table 2:**
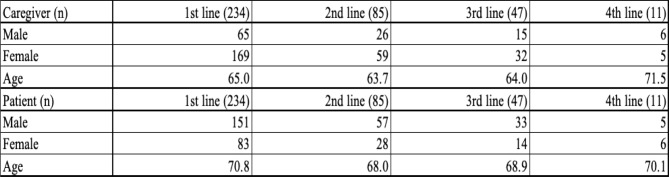
Caregivers and Patients

Legend: Includes age, gender, and living arrangements for both patients and their primary caregivers

**Table 3:**
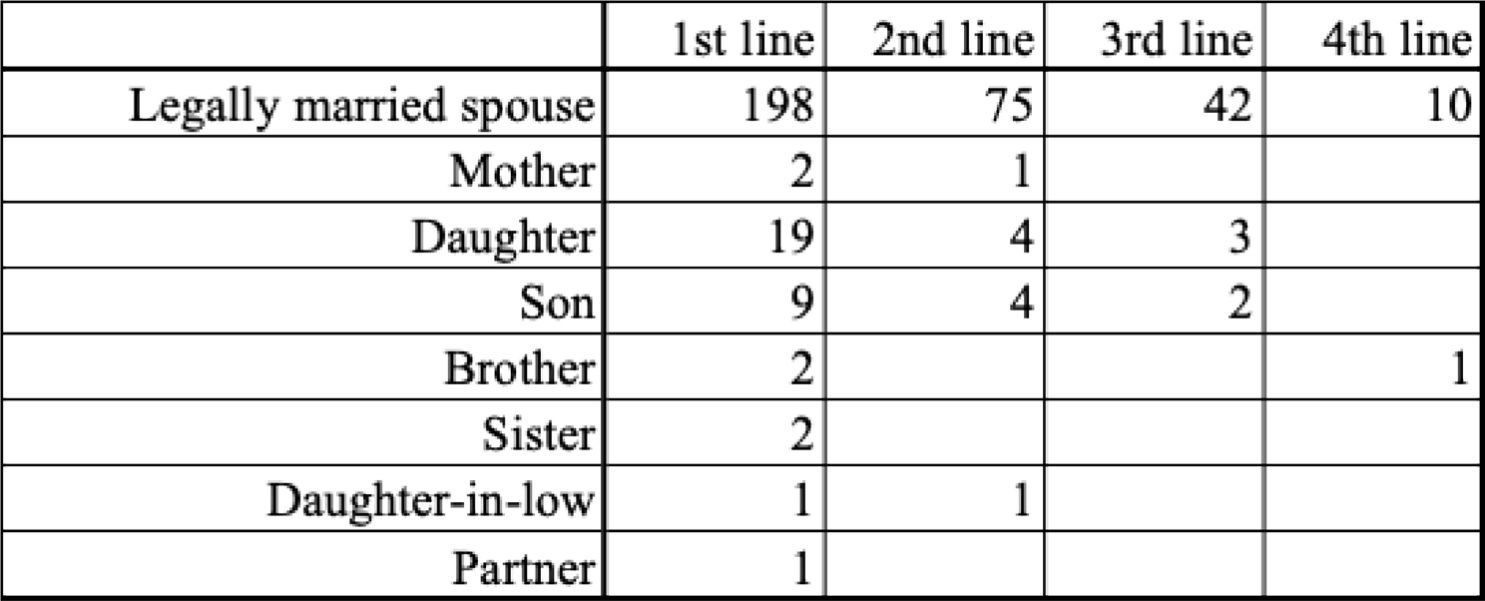
Caregiver relationship

Legend: Shows the legal or familial relationship; over 87% were legal spouses

**Fig. 2:**
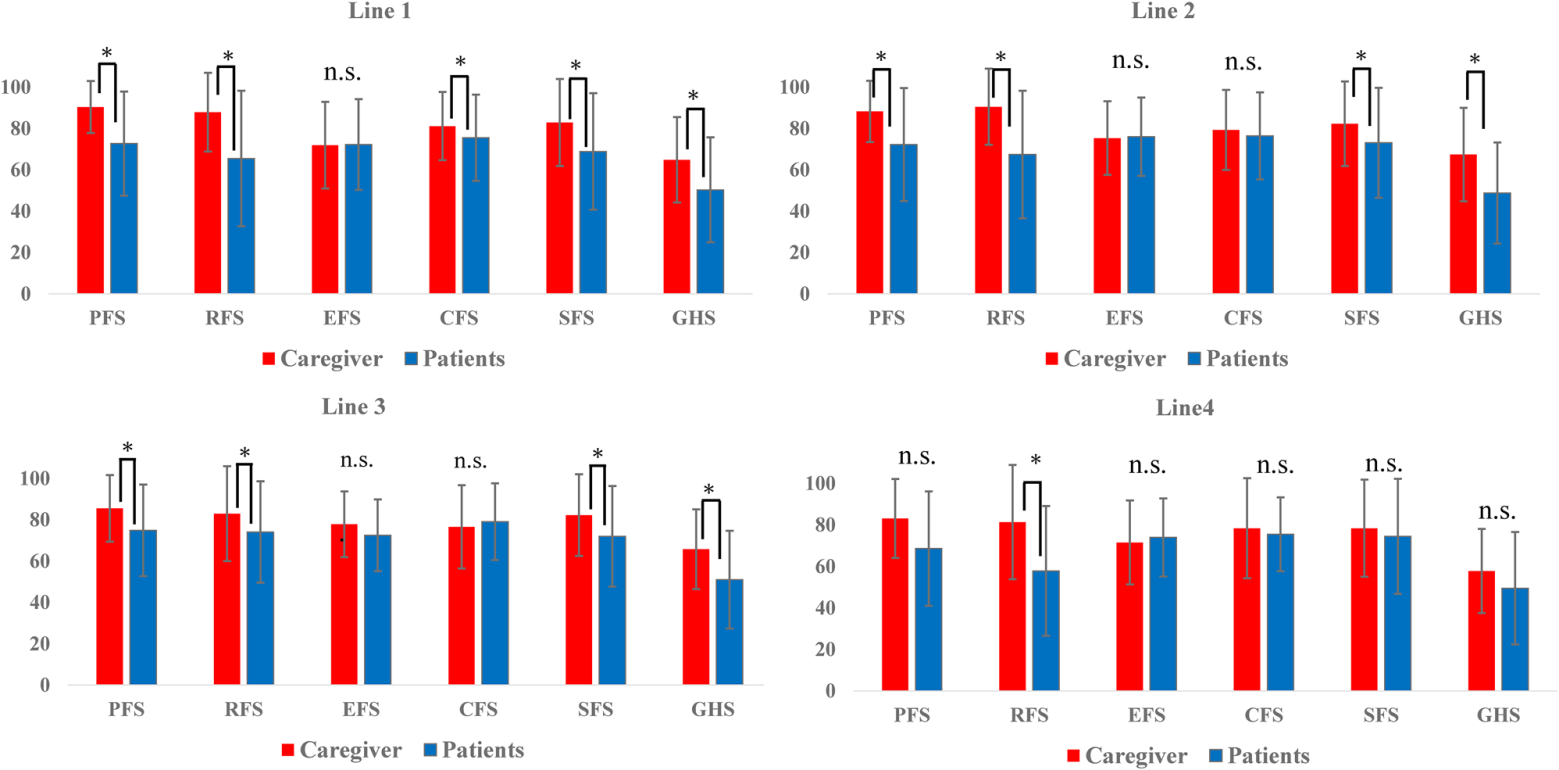
Comparison of QOL Scores Between Patients and Caregivers by Treatment Duration. Comparison stratified by duration of treatment: < 6 months, < 12 months, and > 18 months

Analysis by Treatment Line (Fig. 2, Table 4).

**Table 4:**
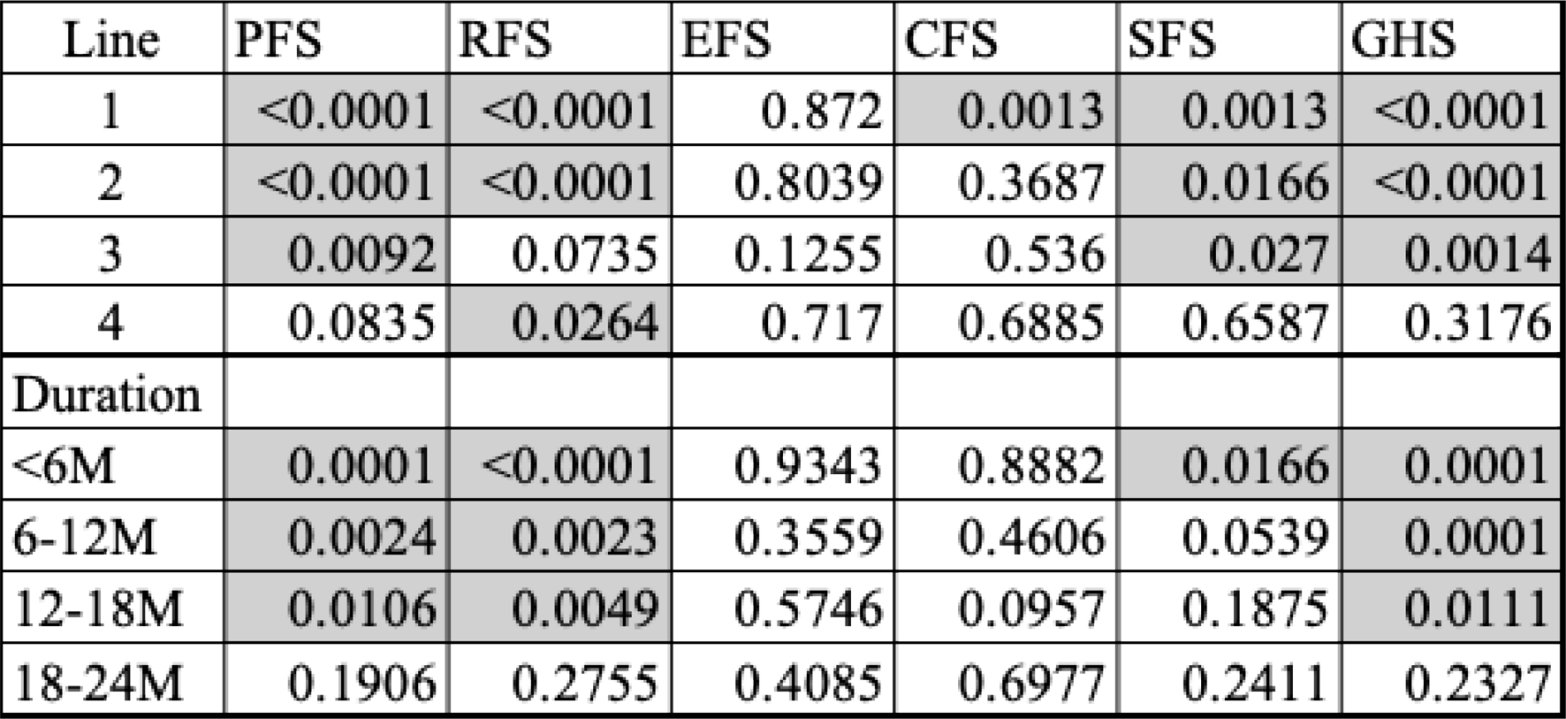
Variation in P-values for Caregiver-patient QOL score differences by treatment line and duration

Legend: Shows p-values from paired t-tests comparing patient and caregiver QOL scores across each treatment line and treatment duration categories

No significant differences in scores on the EFS were found between patients and caregivers at any treatment line. Differences in the CFS were no longer evident after the second-line treatment. By the time of the fourth-line treatment, there were no significant differences between patients and caregivers on any of the QOL scales, except for the RFS (paired t-test, p < 0.05).

**Fig. 3:**
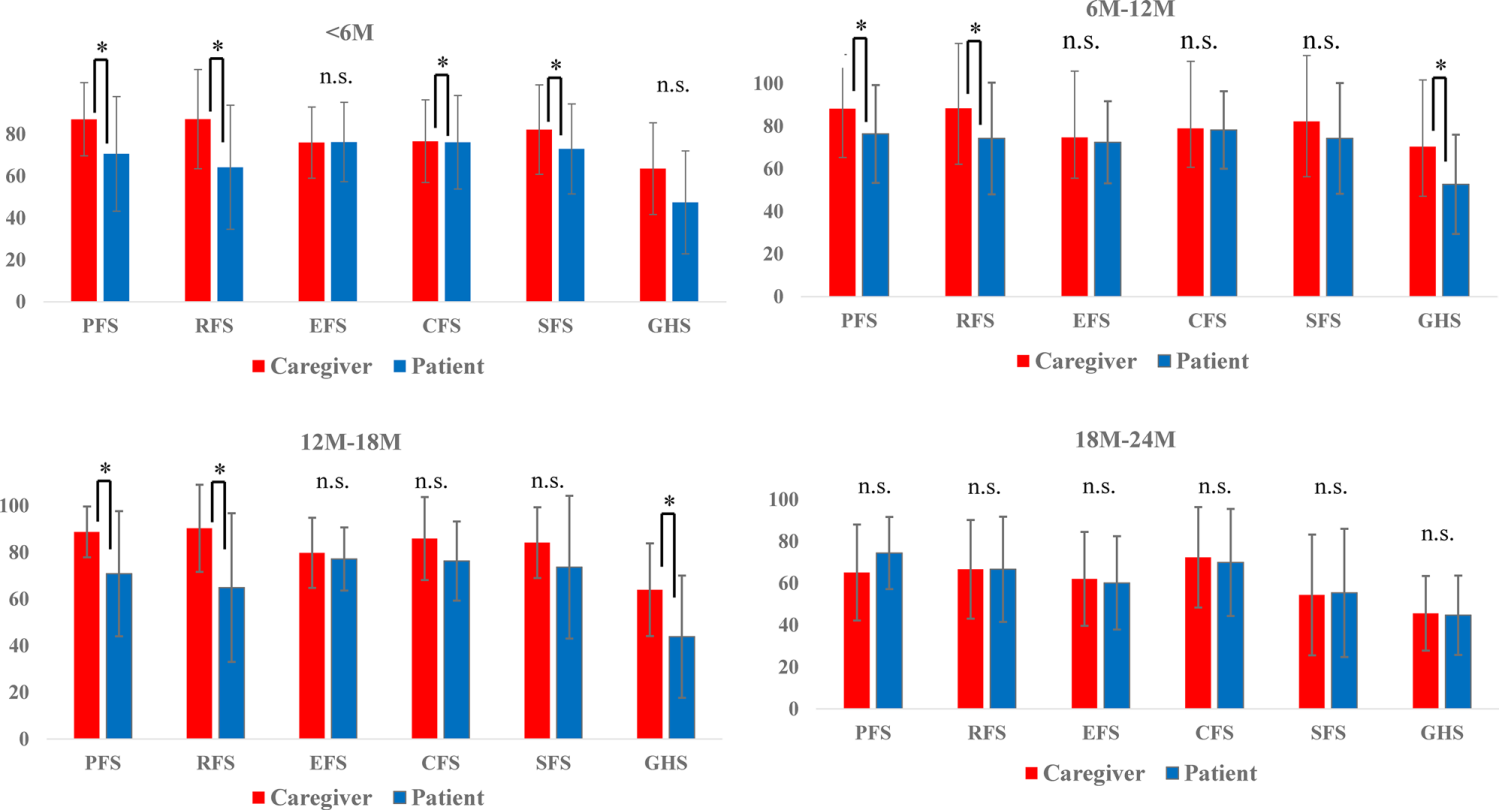
Prevalence of Caregiver-Reported Symptoms by Treatment Line. Proportion of caregivers experiencing specific symptoms such as fatigue, insomnia, and financial difficulty, stratified by treatment line

Analysis by Treatment Duration (Fig. 3, Table 4)

For treatment durations under 6 months, caregivers and CFS scores did not differ significantly from those of the patients, and the caregivers had significantly higher scores in all other areas. For durations under 12 months, no significant differences were observed in the SFS scores. After 18 months, no significant differences were noted between patients and caregivers on any of the scales.

**Fig. 4:**
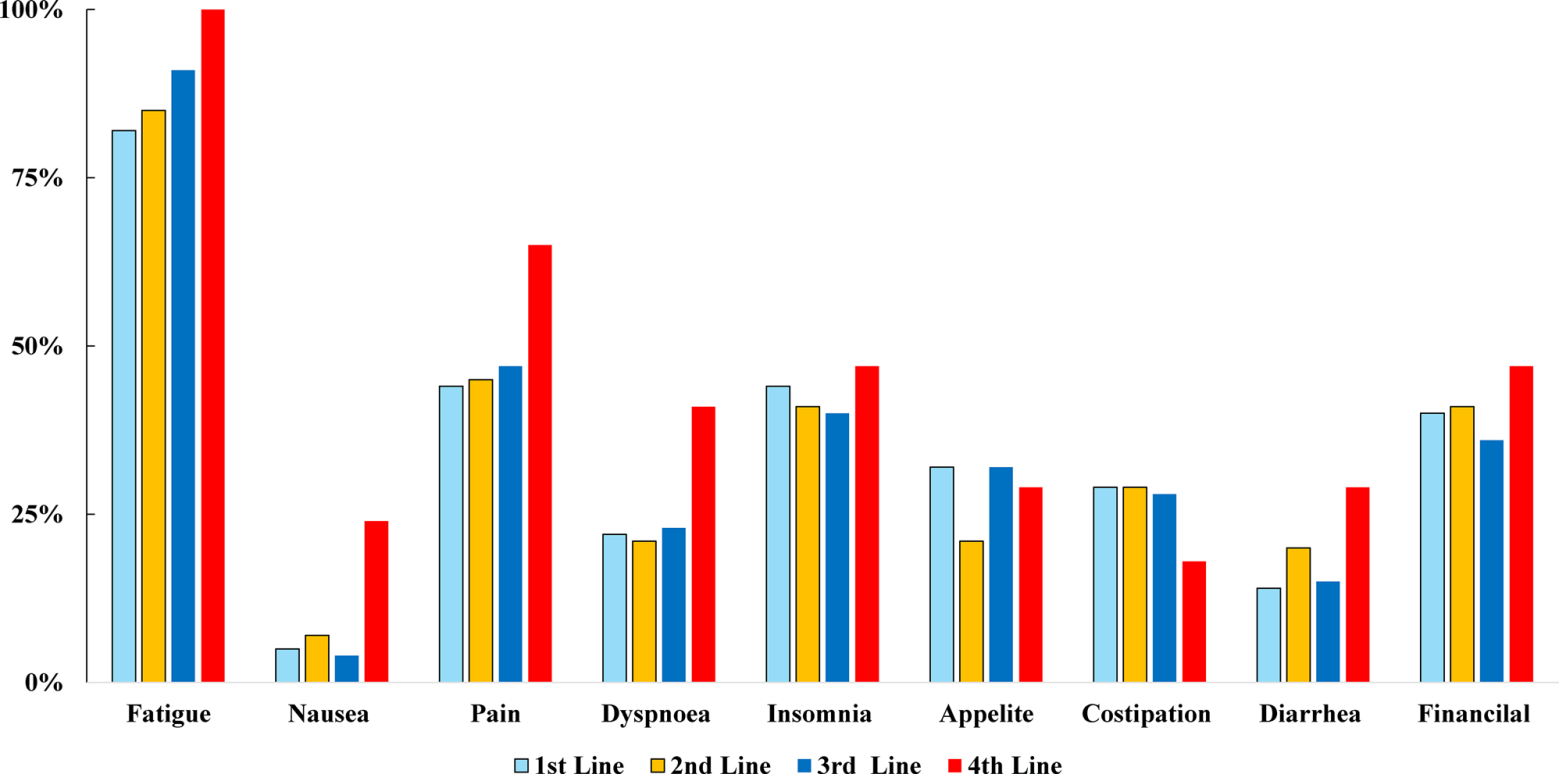
Prevalence of Caregiver-Reported Symptoms by Treatment Duration. Same symptoms as Fig. 3, shown by treatment duration (<6 months, 6–12 months, > 12 months)

**Fig. 5:**
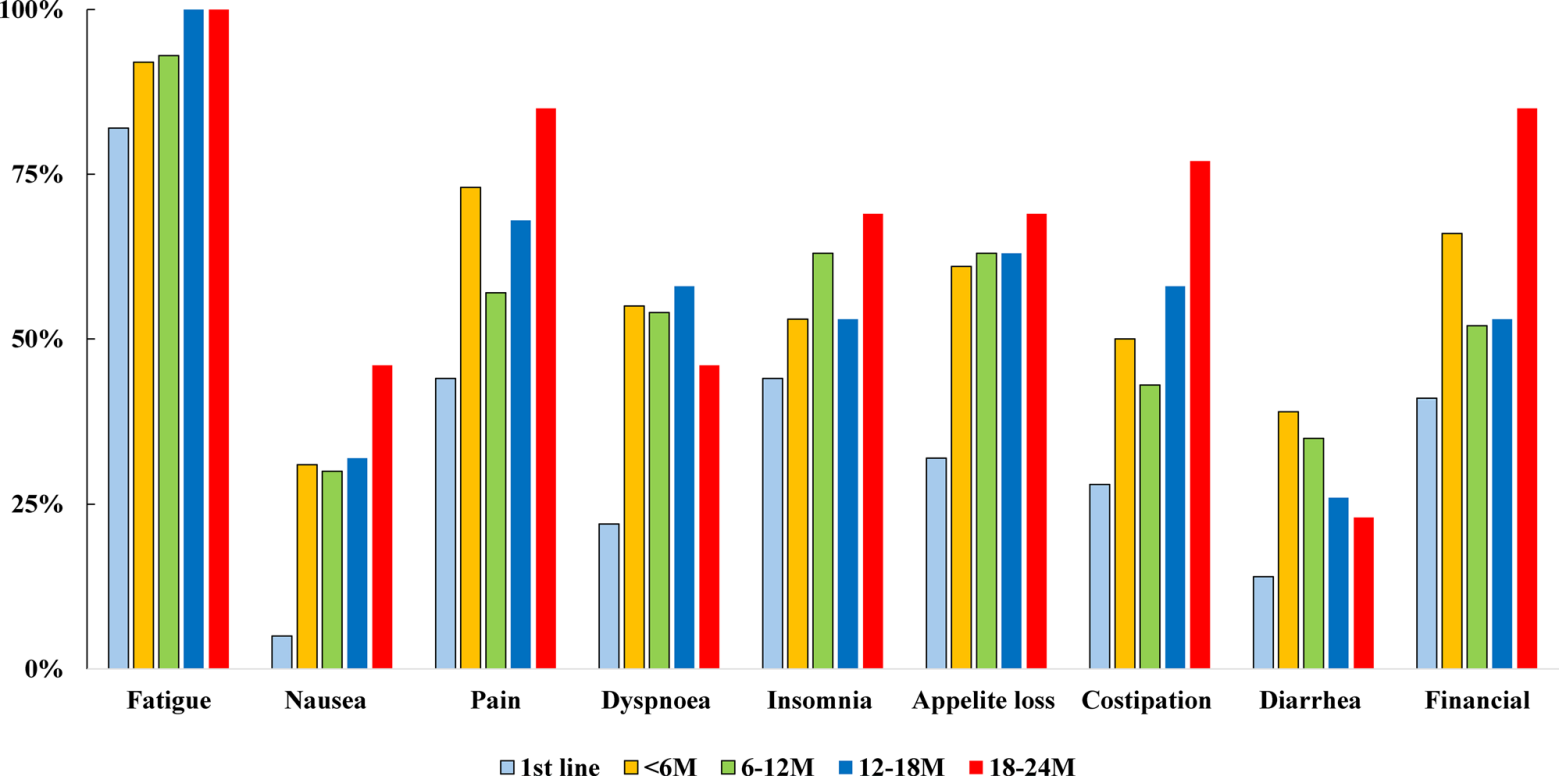
Participant flow. Patient flow diagram showing QOL assessment participation at each treatment line. Both longitudinally followed and newly enrolled patients are indicated

Symptom Scores and Prevalence of Symptoms (Figs. 4 and 5)

The frequency of fatigue among caregivers rose with each treatment line, increasing from 82% before the first-line treatment to 100% before the fourth-line treatment (Fig. 4). Among caregivers with treatment durations of 12 months or longer, 100% of the caregivers reported experiencing fatigue. Furthermore, approximately 40% of caregivers reported symptoms such as insomnia, pain, or financial distress, although no clear patterns related to treatment line or duration were noted (Fig. 5).

Factors Affecting Caregiver QOL: Multivariate Analysis (Table 5)

**Table 5:**
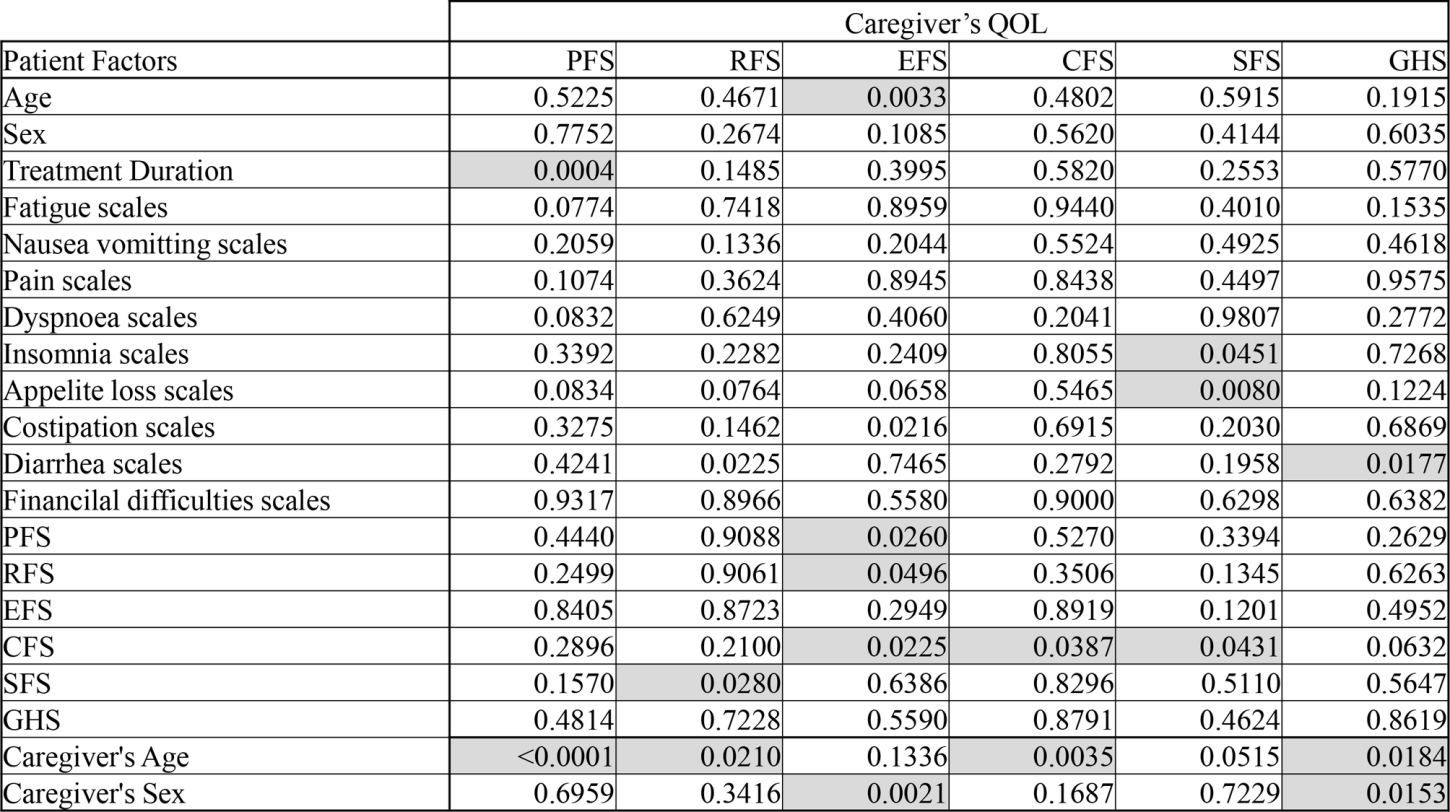
Multivariate analysis of factors affecting caregiver QOL

Legend: Results of multiple regression identifying demographic and patient-related factors affecting caregiver QOL scales

Multivariate linear regression analysis using data from 378 patient-caregiver pairs identified several key factors associated with each caregiver QOL scale.

For the caregiver’s PFS, both caregiver age and treatment duration were significantly related. The caregiver’s RFS was affected by the patient’s SFS and the caregiver’s age. The caregiver EFS was significantly linked to patient age as well as the patient’s PFS, RFS, and CFS, along with caregiver gender, with female caregivers reporting lower scores.

The caregiver’s CFS was influenced by both the patient’s CFS and the caregiver’s age. Similarly, the caregiver’s SFS was significantly impacted by the patient’s CFS. However, the GHS was not significantly linked to any patient- or treatment-related factors but was influenced by caregiver age and gender, with female caregivers showing lower scores.

Regarding patient-reported symptoms, the caregiver SFS was significantly influenced by the patient’s insomnia and loss of appetite, whereas the caregiver GHS was linked to the presence of diarrhea in patients.

## Discussion

Caregivers of palliative chemotherapy patients experience physical, emotional, and social consequences. Previous research has indicated that declining QOL among caregivers results from increasing psychological stress from observing disease progression and treatment, as well as accumulated emotional exhaustion [18, 19]. To maintain or improve caregiver QOL, supportive interventions must be tailored to the treatment phase and the patient condition.

Additionally, caregiver emotional strain may reflect anticipatory grief and existential distress, including loss of purpose and identity. Such distress often precedes patient decline and extends beyond the caregiving role itself

Our findings showed that caregivers’ EFS declined progressively alongside patients as treatment progressed. This pattern extended to CFS and SFS during longer treatment durations or higher treatment lines, eventually leading to deterioration in other QOL domains. These results emphasize the importance of providing early psychological support and establishing social support systems from the beginning of chemotherapy.

Regression analysis showed that lower caregiver EFS was associated with older patient age and lower patient PFS and CFS scores. This suggests that when patients undergo physical or cognitive decline, caregivers are more likely to experience emotional distress. Providing psychological counseling, sufficient information, and access to social services can help alleviate caregiver anxiety and reduce isolation [20]. When needed, psychoeducation and psychiatric consultation should be considered [21, 22].

Declines in caregiver CFS were associated with patient cognitive impairment and caregiver age. In these situations, caregiver stress may manifest as impaired attention, depressive thoughts, anxiety, or sleep-related memory issues [23]. Early intervention through emotional support, support groups, lifestyle guidance, or cognitive training may help caregivers manage multitasking demands and combat social isolation [24, 25]. Because patient’s cognitive decline was associated with deterioration across several caregiver QOL domains, multidisciplinary approaches to support patient cognition may indirectly benefit caregivers [26].

The late-stage decline in caregiver SFS may indicate the growing burdens of increased care demands, reduced social involvement, and heightened loneliness. As the disease advances, it becomes increasingly crucial to provide access to counseling, peer support, and community services.

The high prevalence of caregiver fatigue, sleep disturbances, and financial strain emphasizes the physical challenges involved in home-based care. These investigations highlight the urgent need for systemic, medical, and social interventions tailored to address the caregivers’ needs.

Although the challenges faced by cancer caregivers have been well documented, this study highlights the need for early and ongoing support, particularly for emotional and cognitive aspects, starting at the beginning of treatment [27–29]. Declines in caregiver functioning observed even before treatment suggest psychological distress linked to prognosis and treatment planning. Without timely support, these declines could continue into the terminal phase. To ensure a dignified end-of-life experience for patients and caregivers at home, early integration of social services and multidisciplinary care frameworks is essential.

Future research should examine caregiver responsibilities for other dependents (e.g., children, elderly parents), individual coping mechanisms, and access to formal/informal support networks. These variables may mediate caregiver QOL and inform psychosocial interventions.

### Limitations

Self-selection bias may also have influenced results, as caregivers more interested in health-related topics might have been more likely to participate. Responses may have been subject to social desirability bias due to the self-administered format.

While this study assessed QOL at multiple timepoints, it did not evaluate long-term psychosocial impact beyond the treatment period. Further longitudinal research is warranted.

This study involved a diverse group of patients with cancer and their caregivers, with differences in primary tumor sites and disease stages, which may have contributed to individual variations in QOL outcomes. Moreover, data collection was based on transitions between treatment lines rather than fixed time intervals, potentially increasing variability in the multivariate analyses. Although statistical significance was reached, the overall confidence level in the regression results may be limited.

Economic hardship was reported subjectively rather than measured using objective metrics such as disposable income or out-of-pocket expenses. As a result, individual financial circumstances may have significantly influenced perceptions of burden. Additionally, the average caregiver age in this study was 70 years, which may have affected the findings. Caregiver QOL and symptom scores may have reflected not only caregiving stress but also the physical and psychological challenges associated with aging.

## Conclusion

Advances in cancer treatment have improved survival and QOL for patients. However, caregivers face growing physical and emotional burdens. In this research, caregivers experienced steady declines in emotional and cognitive functioning, regardless of the treatment line or duration, closely reflecting the patient’s condition.

Primary care providers should recognize caregivers as active stakeholders from the outset of chemotherapy, offering tailored support to address emotional and cognitive health. Policymakers should consider embedding caregiver screening and referral systems into oncology care infrastructure. Future trials should evaluate early interventions targeting at-risk caregiver populations to improve QOL trajectories and prevent burnout.

These findings underscore the importance of offering caregivers timely, organized support, similar to that provided to patients, starting early in treatment. This should include sufficient information, psychological counseling, and integration of social support systems. Proactive planning and implementation of comprehensive, caregiver-specific support are essential for maintaining caregiver QOL throughout cancer treatment.

Caregivers should not be invisible stakeholders—they require tailored and timely support strategies to preserve their well-being.

## Data Availability

The datasets used and/or analyzed during the current study are available from the corresponding author on reasonable request.
